# Hybrid Nanomaterial Complexes for Advanced Phage-guided Gene Delivery

**DOI:** 10.1038/mtna.2014.37

**Published:** 2014-08-12

**Authors:** Teerapong Yata, Koon-Yang Lee, Tararaj Dharakul, Sirirurg Songsivilai, Alexander Bismarck, Paul J Mintz, Amin Hajitou

**Affiliations:** 1 Phage Therapy Group, Department of Medicine, Imperial College London, London, UK; 2 Polymers and Composites Engineering (PaCE) Group, Department of Chemical Engineering, Imperial College London, London, UK; 3 National Nanotechnology Center, National Science and Technology Development Agency, Khlong Luang Pathumthani, Thailand; 4 Department of Surgery and Cancer, Imperial College London, London, UK.

**Keywords:** bacteriophage, biomaterials, cancer, cationic polymers, targeted gene transfer, viral particles

## Abstract

Developing nanomaterials that are effective, safe, and selective for gene transfer applications is challenging. Bacteriophages (phage), viruses that infect bacteria only, have shown promise for targeted gene transfer applications. Unfortunately, limited progress has been achieved in improving their potential to overcome mammalian cellular barriers. We hypothesized that chemical modification of the bacteriophage capsid could be applied to improve targeted gene delivery by phage vectors into mammalian cells. Here, we introduce a novel hybrid system consisting of two classes of nanomaterial systems, cationic polymers and M13 bacteriophage virus particles genetically engineered to display a tumor-targeting ligand and carry a transgene cassette. We demonstrate that the phage complex with cationic polymers generates positively charged phage and large aggregates that show enhanced cell surface attachment, buffering capacity, and improved transgene expression while retaining cell type specificity. Moreover, phage/polymer complexes carrying a therapeutic gene achieve greater cancer cell killing than phage alone. This new class of hybrid nanomaterial platform can advance targeted gene delivery applications by bacteriophage.

## Introduction

Successful delivery of gene expression to desired sites *in vivo* following systemic administration will have a major impact on the practice of medicine,^1^ in particular on the advance of gene therapy, genetic imaging, and DNA vaccine applications. Moreover, targeting systemic gene delivery to diseased tissue presents an efficient and safer approach to “theragnostics,” *i.e.*, both gene therapy and genetic imaging combined into one vector system.

Most progress in gene delivery has been made with eukaryotic viruses such as adenovirus, adeno-associated virus (AAV), and lentivirus, which, unquestionably, provide superior gene delivery vectors.^2^ However, systemic administration using these eukaryotic viruses has had limited success due to undesired uptake by the liver and the reticulo-endothelial system, insertional mutagenesis, immunogenicity, pre-existing antibodies, and broad tropism for mammalian tissues.^3^ Numerous materials are being developed to increase gene transfer function. The widely used approaches involved the complexation of naked DNA with cationic polymers or cationic lipids.^4^ Recently, bacteriophages, which are among the most promising new type of biological nanomaterials, have attracted attention as safe and new class of vectors for targeted systemic delivery of transgenes. They have no intrinsic tropism for mammalian cell receptors but can be modified to display tissue-specific ligands on the coat proteins without disruption of their virus structure.^5,6,7,8,9^ Such vectors have many advantages over animal viral vectors and nonviral gene delivery systems due to a number of promising characteristics. Firstly, bacteriophage is a nano-sized natural system capable of efficiently condensing and packaging DNA. The high tolerance for phage coat protein mutations allows insertions of foreign peptides to achieve ligand-directed targeting to the desired cell types and unlike eukaryotic viral vectors, targeting bacteriophage vectors does not require elimination of native tropism.^10^ They are safe, having long been used for both prophylaxis and treatment of bacterial infections, both in adults and children, with no safety concerns being identified.^11^ They have also been approved by the US Food and Drug Administration for use as safe antibacterial food additives.^12^ Large-scale production and purification of phage vectors are simple and economical. Finally, they have a large cloning capacity for insertion of foreign DNA.^13^ We previously introduced an M13 phage-based vector displaying the double cyclic RGD (CDCRGDCFC, RGD4C) ligand to target overexpressed α_v_ integrin receptors in tumors, and incorporating a mammalian transgene cassette flanked by inverted terminal repeats from AAV2. This vector can selectively deliver transgenes to tumors in rodents and pet dogs after intravenous administration, while sparing normal organs.^14,15,16,17,18^ Bacteriophage is thus the first system that has been experimentally shown to offer safe and efficient delivery of transgenes to target tissues after systemic administration *in vivo*. Unfortunately, phage particles are comparatively poor biomaterial vectors, as they have evolved to infect bacteria only. Unlike eukaryotic viruses, they have no intrinsic strategies for delivering genes to mammalian cells.^19,20^ As a result, although phage-derived vectors undoubtedly have great promise, due to this inherent limitation, they need to be improved if they are going to find wide clinical applications.

**Figure 1:**
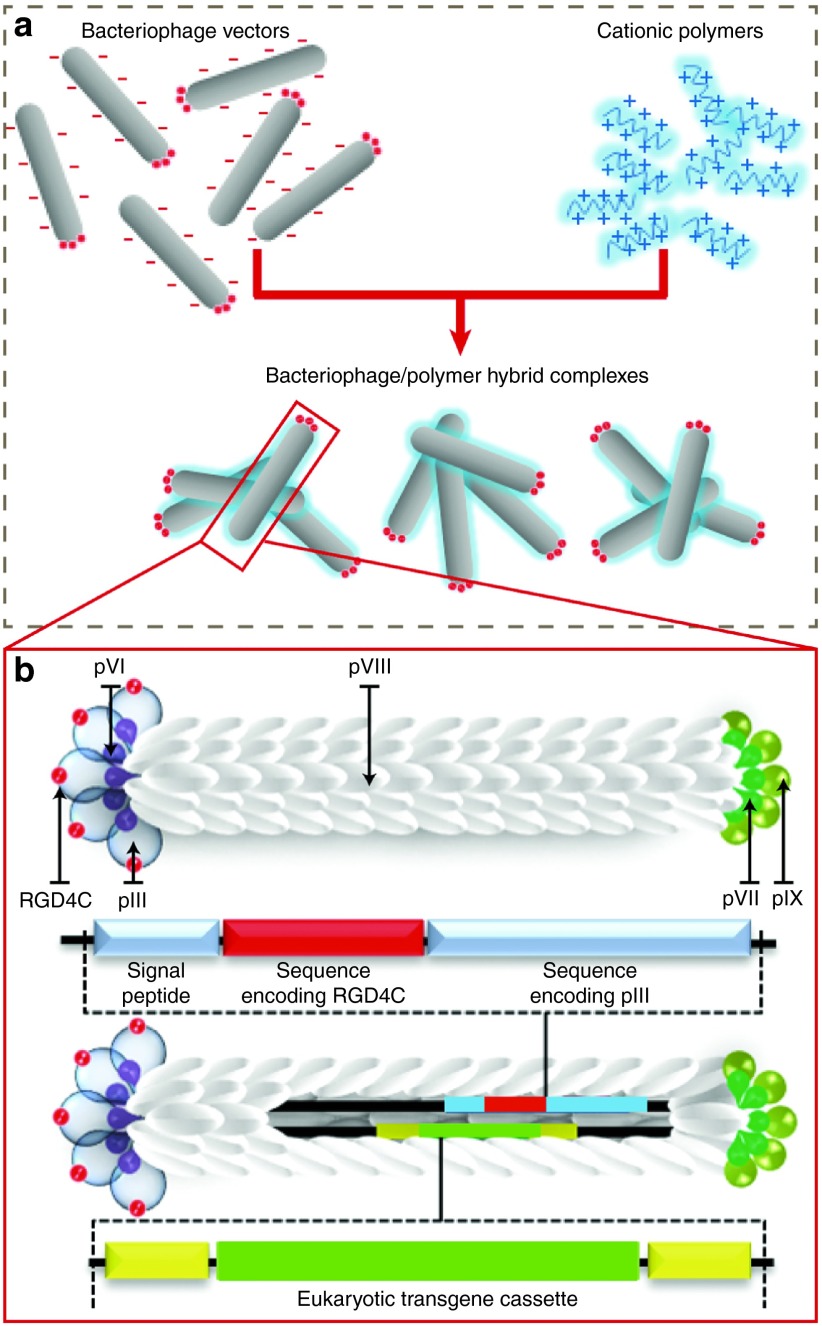
**Schematic diagram of the hybrid bacteriophage/polymer platform.** (**a**) Negatively charged bacteriophage vectors were electrostatically assembled with cationic polymers to form hybrid bacteriophage/polymer complexes. A hybrid vector consists of the filamentous M13 bacteriophage and cationic polymer. The phage serves as a transgene carrier displaying a tumor-targeting peptide on the phage capsid for entry into eukaryotic cells. The polymer counteracts the inherent negative charge of the phage particle. (**b**) Recombinant filamentous bacteriophage with genetically modified genome.

As a proof-of-concept study, we report a hybrid platform of phage and synthetic materials. They are self-assembled complexes consisting of a recombinant M13 bacteriophage displaying the RGD4C-targeting ligand and containing a eukaryotic transgene cassette, coupled with a synthetic cationic polymer material (**Figure 1**). We have investigated their physical and chemical properties (surface chemistry, electrokinetic behavior, size, and morphology), and their biological activity (transgene expression, cytotoxicity, cell–vector interaction, and delivery mechanisms). Integration of phage with polymers successfully generated positively charged, large-sized particles with increased potential for binding to the surface of eukaryotic cells, better buffering capacity suggesting enhanced endosomal escape and subsequently improved gene transfer efficiency. Importantly, gene delivery by the hybrid vector remained targeted and specific, inducing cancer cell killing. We believe this innovation represents a major advance in phage-mediated gene transfer with potential for clinical applications.

## Results

### Integration of phage with cationic polymers boosts gene transfer to mammalian cells by phage-based vectors

**Figure 2:**
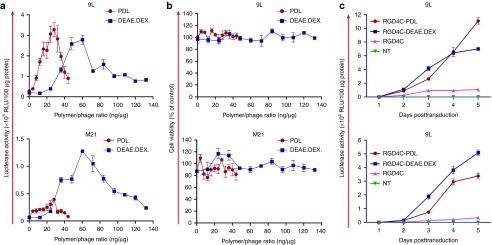
**Characterization of tumor cell transduction by the hybrid phage/polymer. (a**) Optimization of polymer types and concentrations. M21 and 9L cells were treated with RGD4C-phages carrying the *Luc* transgene premixed with increasing concentrations of poly-d-lysine (PDL) and DEAE.DEX and *Luc* gene expression was measured using the luciferase assay at day 3 post-transduction. (**b**) Cytotoxicity of the RGD4C-phage complexed with increasing concentrations of cationic polymers in both M21 and 9L cell lines. Cell viability was measured using the CellTiter-Glo cell viability assay, at 48-hour-post transduction. The cell viability rate (%) was calculated as percentage of control ([*A*]_test_/[*A*]_control_ × 100) (*n* = 3). (**c**) Kinetics of *Luc* gene expression following transduction of M21 and 9L cells with the RGD4C-phage premixed with PDL or DEAE.DEX (RGD4C-PDL and RGD4C-DEAE.DEX, respectively), RGD4C-phage alone (RGD4C), or non-targeted phage (NT). The luciferase assay was performed daily over a time course of 5 days after vector transduction.

We sought to assess whether the efficiency of gene delivery by the RGD4C-phage to eukaryotic cells can be improved if phage viral particles are integrated with cationic polymers. We therefore studied the efficacy with which RGD4C-phage/polymer complexes transduce human M21 melanoma cells, which are known to express high levels of α_v_ integrin receptors for the RGD4C ligand.^17,21^ To rule out the possibility that the observed effects are not cell or species specific, we also assessed the efficacy on the rat 9L glioblastoma cells, which have previously been shown to be transduced by the RGD4C-phage.^19,22^ We first sought to determine the optimal ratio of two cationic polymers (poly-d-lysine (PDL) and DEAE-DEX) and RGD4C-phage using RGD4C-phage vector carrying the firefly luciferase (*Luc*) reporter gene. Quantification of luciferase activity in 9L and M21 cells at 72-hour post-cell transduction showed that *Luc* gene expression by the RGD4C-phage dramatically improved with increased concentrations of PDL and DEAE.DEX polymers (**Figure 2a**), as compared with RGD4C-phage alone (0 μg/ml of polymer). Maximum gene transfer levels were achieved in both M21 and 9L cells at polymer/phage ratios of 30 ng/μg for PDL and 60 ng/μg for DEAE.DEX, respectively, after which a gradual decrease in *Luc* gene expression occurred (**Figure 2a**). To determine whether the decreased transgene expression at high amounts of cationic polymers was associated with PDL and DEAE.DEX cytotoxicity, we performed cell viability assays and showed that this range of polymer concentrations was not associated with any toxic effects (**Figure 2b**).

Next, we used the previously established optimal ratios of polymer and phage to assess the efficacy of gene transfer by the hybrid RGD4C-phage/polymer complexes over a period of 5 days following transduction of M21 and 9L cells (**Figure 2c**). Four different vector systems were investigated: non-targeted phage (NT), targeted RGD4C-phage (RGD4C) displaying the tumor-targeting ligand on pIII minor coat protein, RGD4C-phage complexed with PDL (termed RGD4C-PDL), and RGD4C-phage complexed with DEAE.DEX (termed RGD4C-DEAE.DEX). Considerable increase in expression of the *Luc* transgene was detected in both M21 and 9L cells transduced with the hybrid RGD4C-PDL and RGD4C-DEAE.DEX phage/polymer complexes at day 5 post-transduction (**Figure 2c**). This *Luc* gene expression increased rapidly over time, whereas gene expression remained low in cells transduced by the RGD4C-phage, and none was detected in cells incubated with a control NT phage. For instance, at day 5 post-transduction, treatment with RGD4C-PDL and RGD4C-DEAE.DEX phage/polymer resulted in ~10.3- and ~6.6-fold increase in *Luc* gene expression in 9L cells and ~10.0- and ~15.0-fold in M21 cells, respectively, compared with RGD4C-phage alone (**Figure 2c**).

Next, to further explore the superiority of the RGD4C-phage vector when combined with cationic polymers, we assembled a panel of cancer and normal cell lines. The human LN229 and SNB19 glioma cells were incubated with vectors bearing the *Luc* reporter transgene. Marked and dose-dependent increase in gene delivery was detected with RGD4C-PDL or RGD4C-DEAE.DEX compared with uncomplexed RGD4C-phage (**Supplementary Figure S1a**). Similar effects of the DEAE.DEX polymers were also observed in the rat C6 brain tumor cells (**Supplementary Figure S1a**). These data were confirmed by using vectors carrying the green fluorescent protein (*GFP*) reporter gene (**Supplementary Figure S1b**).

Finally, we also evaluated efficacy in a nontumor cellular model using the human embryonic kidney (HEK293) cell line. These cells have extensively been used as a standard *in vitro* model to characterize cell transduction by RGD4C-phage vectors since they express high levels of α_v_β3 and α_v_β5 integrins.^15,16^ Thus, HEK293 cells were treated with vectors bearing the *Luc* or *GFP* reporter transgenes. Quantitative analysis of *Luc* activity at day 3 post-vector transduction showed that *Luc* gene expression by the RGD4C-DEAE.DEX or RGD4C-PDL was significantly enhanced with increased concentrations of DEAE.DEX and PDL polymers (**Supplementary Figure S2a**), as compared with RGD4C-phage alone (0 μg/ml of polymer). Maximum transduction efficiency was achieved at optimal polymer/phage ratios of 250 ng/μg for DEAE.DEX and 125 ng/μg for PDL, followed by a gradual decrease in *Luc* gene expression (**Supplementary Figure S2a**). Importantly, no effect on HEK293 cell viability was induced by this range of polymer concentrations (**Supplementary Figure S2b**). These data were confirmed with microscopic imaging of GFP expression in HEK293 cells, revealing increase in GFP expression in those treated with RGD4C-phage/polymer complexes (**Supplementary Figure S3**). No GFP expression was detected in cells treated with the control NT phage. Altogether, these data validate that the integration of cationic polymers with bacteriophage boosts gene transfer efficiency.

### Characterization of the hybrid phage/polymer complexes

**Figure 3:**
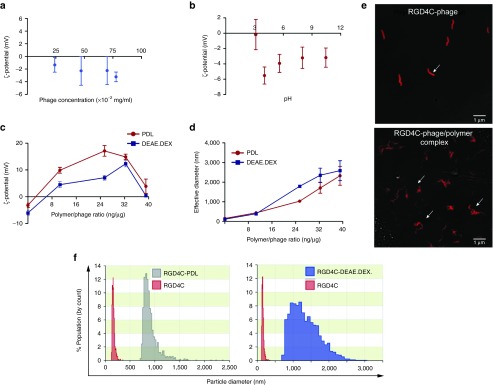
**Physical characterization of phage/polymer complex.** (**a**) The ζ-potential of phage was measured by Zetasizer when varying phage titer. (**b**) ζ-potential of phage as a function of pH. The isoelectric point of phage particles was determined at the cross point when the ζ -potential becomes zero. (**c**) ζ -potential analysis of phage following various and increasing polymer (DEAE.DEX or PDL) concentrations. (**d**) Average size measurements of phage coated with polymers at increasing polymer concentrations as indicated. (**e**) Appearance of phage/polymer complexes. Morphology of hybrid complexes and phage vectors alone were analyzed using confocal microscopy. Phage particles were stained for immunofluorescence using anti-phage primary and goat anti-rabbit AlexaFluor-647 secondary antibodies. (**f**) Size distribution of the RGD4C-phage and RGD4C-phage complexed with polymers (RGD4C-PDL or RGD4C-DEAE.DEX).

To gain insight into the characterization of the hybrid phage/polymer complexes, we sought ways to explore the electrostatic charge on the phage capsid. We first investigated the charge characteristics of the bacteriophage viral particles by measuring their ζ-potential using electrophoresis (**Figure 3a**). As expected, we found that bacteriophage is negatively charged at physiological pH (**Figure 3a**). The data also indicate that RGD4C-phage possesses an acidic surface, with an isoelectric point of pH = 3 as determined from ζ-potential = *f*(pH) (**Figure 3b**). Next, we analyzed the ζ-potential of bacteriophage vectors following hybridization with cationic polymers PDL and DEAE.DEX (**Figure 3c**). We found that addition of increasing concentrations of both cationic polymers PDL and DEAE.DEX resulted in gradual shift of the ζ-potential from a negative value for unmodified bacteriophage to a positive value for the phage complexed with polymers to reach maximum positive values after which the ζ-potential started to drop significantly. For instance, incorporation of 25-ng PDL with 1 μg of phage resulted in shifting the ζ-potential from −5 mV to +15 mV, after which the ζ-potential dropped sharply toward a zero value. This increase in ζ-potential can be attributed to surface adsorption of the polymer counteracting the inherent negative charge of the bacteriophage particles.

Next, we conducted a second set of experiments to assess whether addition of cationic polymers leads to aggregation of the phage viruses resulting in the formation of large size particles. Size measurements of phage/polymer complexes revealed that increasing the polymer concentrations resulted in gradual increase in the average size of hybrid complexes (**Figure 3d**). These results were confirmed by confocal microscopy and anti-phage staining showing the morphology of uncomplexed phage as single filamentous particles, whereas the addition of cationic polymers resulted in aggregation of the phage particles (**Figure 3e**). Moreover, size distribution of the RGD4C-phage and RGD4C-phage/polymer complexes revealed marked heterogeneity in size and populations of the RGD4C-PDL and RGD4C-DEAE.DEX complexes (**Figure 3f**).

Finally, we investigated whether complex formation was required prior to treatment of cells. Thus, 9L cells were first treated with cationic polymers alone followed by addition of the RGD4C-phage. We found that separate treatments of cells starting with cationic polymers and then with RGD4C-phage had no significant effect on gene delivery efficacy (**Supplementary Figure S4**). These data suggest that formation of the phage/polymer complex, prior to incubation with cells, is required to improve transduction efficiency.

Altogether, these results indicate that negatively charged phage particles are physically incorporated with cationic polymers to form large complex aggregates.

### Cell surface accessibility and internalization of the phage/polymer

**Figure 4:**
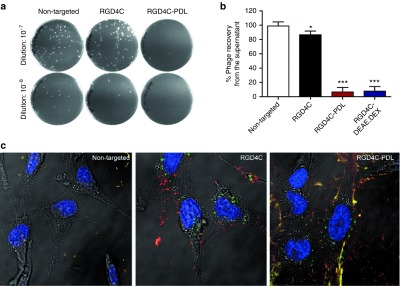
**Characterization of the hybrid phage/polymer cell surface accessibility.** Evaluation of the phage/polymer cell surface accessibility by quantifying the free cell-unbound phage in the external fluid phase above the adherent cell layer by infection of host bacteria. (**a**) Representative plates showing bacterial colonies generated by phage recovered from the supernatant of transduced cells. (**b**) Quantitative analysis of the recovered phage following bacterial colony counting. (**c**) Confocal fluorescent microscopic images of 9L cells following treatment with different vectors. Cells were first immunofluorescent stained for extracellular phage using an anti-phage primary and goat anti-rabbit AlexaFluor-594 secondary (*red*) antibodies prior to permeabilization and staining for intracellular phage using the same primary and goat anti-rabbit AlexaFluor-488 secondary (*green*) antibodies.

We also investigated the phage/polymer cell surface accessibility to determine whether gene delivery efficiency by the RGD4C-phage is limited by inefficient access to the negatively charged cell surface. We therefore carried out a supernatant-depletion assay, where the free cell-unbound phage in the external fluid phase above the adherent cell layer was quantified by infection of host bacteria followed by colony counting (**Figure 4a**). A large amount of free phage particles (90% of input phage particles) were recovered from the supernatant of cells treated with the RGD4C-phage vector (**Figure 4b**), showing that only a small fraction (10% of input phage) was bound to the cell surface. By contrast, very little phage (6%) was recovered from the supernatant of cells incubated with RGD4C-PDL and RGD4C-DEAE.DEX phage/polymer complexes, indicating that most of the phage (94%) was bound to the surface of cells (**Figure 4b**). No phage depletion was observed in the supernatant of cells treated with the control NT phage (**Figure 4b**). Confocal microscopic imaging following immunofluorescence with an anti-phage antibody revealed greater cell surface localization of the RGD4C-phage/polymer aggregates than the uncomplexed RGD4C-phage alone (**Figure 4c**). No phage was observed on cells incubated with the control NT phage (**Figure 4c**). These data strongly suggest that incorporation of phage into a cationic complex increases phage accessibility to the cell surface.

Finally, internalization assays revealed that incorporation of phage with the cationic polymers PDL or DEAE.DEX does not increase cellular entry of the RGD4C-phage (**Supplementary Figure S5)**.

### Buffering capacity of the hybrid phage/polymer complexes

**Figure 5:**
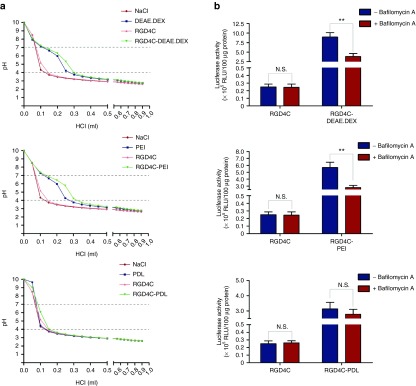
**Endosome buffering capacity of phage/polymer complexes. (a**) Acid–base titration curve of phage/polymer complexes. To assess the proton buffering capacity, the phage complexed with DEAE.DEX, PEI or PDL was dissolved to their optimized ratios in water and adjusted to pH 10. Individual polymers and phage alone were also included in this experiment. HCl was used to titrate the solutions to pH 3. The pH was measured by the pH meter. Titration curve of NaCl was used as a control. Dashed lines represent the typical pH range in the endosome (pH 7.0–4.0). (**b**) Effect of bafilomycin A1 on transduction efficiency of phage/polymer complexes. HEK293 cells were treated with 25 µmol/l bafilomycin A1for 1 hour, and then transduced with the RGD4C-DEAE.DEX, RGD4C-PEI, RGD4C-PDL phage/polymer complexes or control RGD4C-phage. After 3 days, cells were analyzed for *Luc* expression. N.S., nonsignificant.

Endosomal escape is an important factor to be considered for the design of gene delivery vectors. This mechanism is associated with the buffering capacity of gene vectors within the pH range, in which vectors traffic from the extracellular environment into acidified endosomal compartments. Polycations with high buffering capacity can mediate efficient escape from the endosome to the cytosol triggered by the acidic endosome via a mechanism termed the “proton sponge effect.”^23^ A buffering capacity should allow the complexes to absorb protons pumped into the endosomes resulting in an influx of Cl^−^ ions to prevent the build-up of a charge gradient. This influx of both protons and Cl^−^ ions increases the osmolarity of the endosomes leading to osmotic swelling, subsequent endosomal destabilization and release of their contents into the cytoplasm. We previously identified the endosomal-lysosomal degradative pathway as an intracellular barrier to RGD4C-phage-derived particles, which are sequestered and degraded within the lysosomes, reducing their ability to deliver genes to mammalian cells.^20^ It was reported that cationic polymers increase the gene transfer efficiency of nonviral vectors due to their ability to induce endosomal escape.^23^ Therefore to assess the buffering capacity of the phage/polymer complexes, we performed acid–base titrations to compare the polymer, RGD4C-phage alone and the hybrid complexes with a sodium chloride (NaCl) control solution (**Figure 5a**). Moreover, we extended the list of cationic polymers to include the poly(ethyleneimine) (PEI), since this polymer has been reported for its buffering capacity and potential to enhance endosomal escape of delivery vectors.^24^

We found that both complexes RGD4C-DEAE.DEX and RGD4C-PEI have high buffering capacity similar to DEAE.DEX and PEI polymers, respectively (**Figure 5a**), and required additional HCl, compared to control NaCl, to lower the pH from 7.0 to 4.0 (**Figure 5a**). The uncomplexed RGD4C-phage vector displayed a titration curve similar to NaCl and therefore has no buffering capacity (**Figure 5a**). An important finding was that RGD4C-PDL complex had no buffering capacity compared with RGD4C-DEAE.DEX and RGD4C-PEI (**Figure 5a**). This result is consistent with the *Luc* gene expression analyses demonstrating higher transduction efficacies of RGD4C-DEAE.DEX and RGD4C-PEI than RGD4C-PDL (**Figure 5b**). These data provide strong evidence that the buffering capacity of the RGD4C-DEAE.DEX and RGD4C-PEI hybrids contributes to their gene delivery efficiency. To confirm these findings, we performed transduction experiments in the presence of bafilomycin A1, a specific inhibitor of the vacuolar ATPase proton pump that prevents endosomal escape.^20^ The results showed a significant decrease in transduction efficiencies of RGD4C-DEAE.DEX and RGD4C-PEI in the presence of bafilomycin A1; in contrast, there was no significant effect on RGD4C-PDL (**Figure 5b**). These data give further evidence to an additional mechanism of improved gene delivery by the RGD4C-DEAE.DEX and RGD4C-PEI phage/polymer complexes by facilitating escape from the endosome-lysosome degradative pathway.

### The phage/polymer hybrid complexes retain targeting and specificity of gene transfer

**Figure 6:**
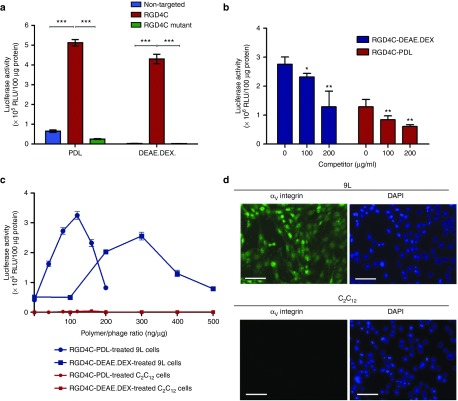
**Evaluation of the specificity of cell transduction by the targeted RGD4C-phage/polymer complexes. (a**) Targeted gene transfer to 9L tumor cells by the RGD4C-phage/polymer was compared with that of negative phage/polymer controls (non-targeted or mutated). **(b**) Inhibition of transduction efficiency of the hybrid RGD4C-PDL and RGD4C-DEAE.DEX complexes by an RGD4C-expressing phage. **(c**) Targeted gene transfer by the RGD4C-phage/polymer complexes was assessed in the normal C_2_C_12_ myoblast cell line, using increasing concentrations of the PDL or DEAE.DEX cationic polymers. (**d**) Expression of the α_v_ integrin receptor was investigated in 9L tumor cells and C_2_C_12_ myoblast cell line with anti-integrin primary and AlexasFluor-488 secondary antibodies.

We want to confirm that the targeting properties of the RGD4C-phage vector remain intact in the phage/polymer complex and that transduction of cells is specific and mediated by binding of the RGD4C ligand to the α_v_ integrin receptors. Therefore, cell transduction efficiency of the RGD4C-phage/polymer complex was compared with that of complexes between polymer and either NT phage or phage displaying a mutant version of the RGD4C sequence (RGE4C, D–E), previously reported as NT controls for the RGD4C-phage.^14,16^ As shown in **Figure 6a**, *Luc* gene expression in cells treated with the RGE4C-phage/polymer was nonsignificant and comparable to that of NT phage/polymer complex.

Next, to confirm specificity, we carried competition experiments by incubation with a phage lacking a gene expression cassette, but expressing the RGD4C peptide on the capsid, because use of significant excess of the soluble RGD ligand causes cell toxicity. The data showed that the RGD4C-expressing phage inhibits gene delivery by the targeted RGD4C-PDL and RGD4C-DEXAE.DEX phage/polymer complexes in a dose-dependent manner (**Figure 6b**).

Finally, to prove the ability of the RGD4C-phage/polymer complexes to target gene delivery to α_v_ integrin-expressing cells specifically, but not cells lacking the α_v_ integrin receptors, we compared transduction efficiencies between the 9L tumor cells and the normal C_2_C_12_ myoblast mouse cell line using RGD4C-PDL and RGD4C-DEAE.DEX complexes containing increasing concentrations of either PDL or DEAE.DEX cationic polymers (**Figure 6c**). While the RGD4C-phage/polymer complexes reproduced the expected pattern of *Luc* transgene expression in 9L cells, no transgene expression was induced in C_2_C_12_ cells by any concentration of either PDL or DEAE.DEX polymers (**Figure 6c**). We also confirmed the expression of the α_v_ integrin receptors on the 9L tumor cells, in contrast to the C_2_C_12_ myoblast cells lacking this integrin (**Figure 6d**). These findings confirm that gene delivery by the RGD4C-phage/polymer complex is targeted, specific, and dependent on α_v_ integrin receptors.

### Enhancement of herpes simplex virus thymidine kinase (HSVtk)/ganciclovir (GCV)-mediated cell death by the hybrid phage/polymer complex

**Figure 7:**
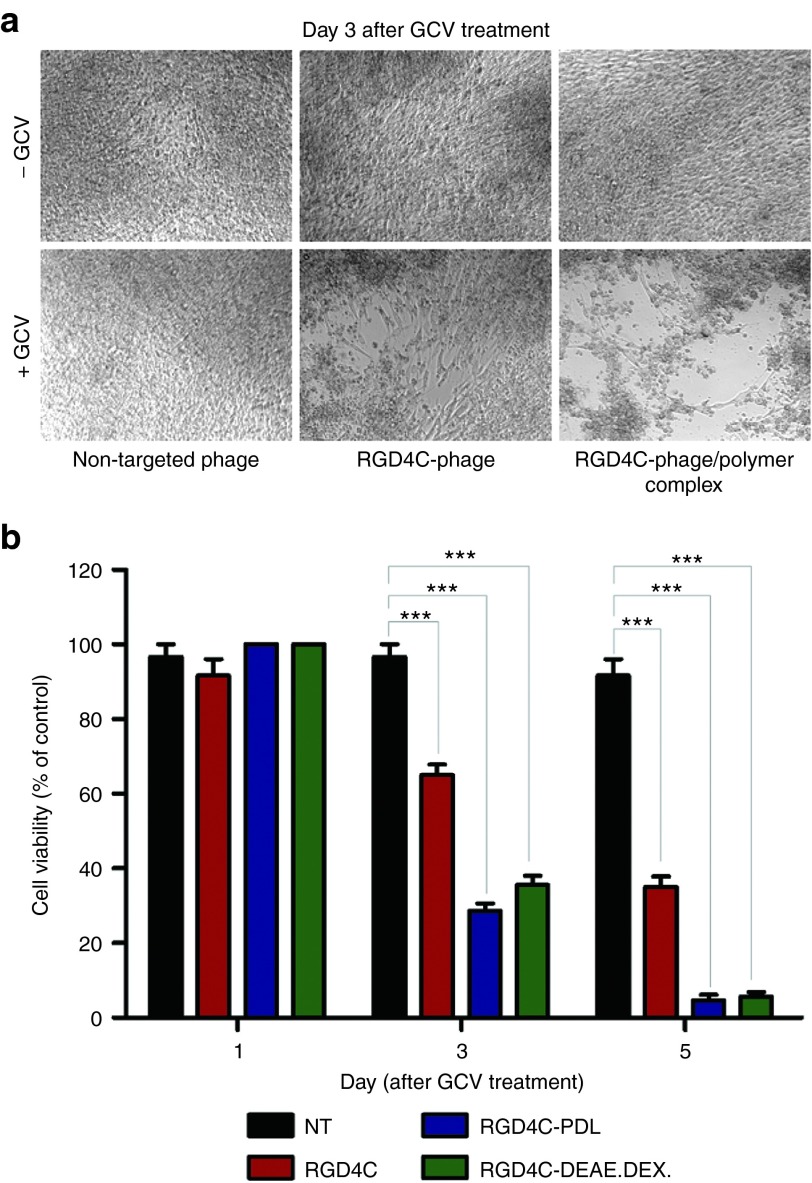
***HSVtk*/GCV-mediated cancer cell death by the hybrid phage/polymer complex. (a**) Morphological characteristics of 9L cells following *HSVtk*/GCV therapy, as visualized with a bright field microscope. Confluent cells in the absence of GCV or cells treated with non-targeted phage and GCV show normal morphology. (**b**) Evaluation of tumor cell killing by the RGD4C-phage/polymer complex, RGD4C-phage, and the non-targeted phage in 9L cells at days 1, 3, and 5 post**-**GCV treatment. Cell viability was determined by the CellTiter-Glo cell viability assay.

After establishing the integration of cationic polymers with bacteriophage vectors substantially increases their gene transfer efficacy, we sought to assess the effect of polymer on a specific clinical application of phage, namely gene therapy. To test the efficacy of tumor cell killing of the RGD4C-phage/polymer complex, we constructed RGD4C-phage vector carrying the *HSVtk* gene. When combined with the GCV prodrug, this gene can serve as a suicide gene. We chose to conduct our experiments in the 9L glioblastoma model as this tumor type is highly aggressive and remains a major clinical challenge. We compared the RGD4C-phage vector alone with the complexes RGD4C-PDL and RGD4C-DEAE.DEX. *HSVtk* suicide gene therapy was induced at day 3 post-transduction by daily treatment with GCV for 5 days. Morphological characteristics of 9L cells 3 days post-GCV treatment were visualized by brightfield microscopy. **Figure 7a** shows normal morphology of confluent cells in all treatments in the absence of GCV and in cells treated with a combination of NT phage and GCV. There was a significant increase in cells detaching from the substrate by the RGD4C-phage/polymer, upon GCV treatment, as compared with the uncomplexed RGD4C-phage. Cell viability assays were measured over a time course of 1, 3, and 5 days post-GCV treatment (**Figure 7b**). The results show greater tumor cell killing by the RGD4C-phage/polymer than RGD4C-phage alone at both days 3 and 5 post-GCV (**Figure 7b**). No cell death was detected in cells treated with NT phage (**Figure 7b**). It is important to note that the *HSVtk/GCV* approach elicits a bystander effect which can take over beyond day 5, resulting in increased cell killing by the RGD4C-phage alone. Thus, the cell killing results are partially proportional to the transduction efficiency.

### Evaluation of efficacy in a three-dimensional (3D) multicellular tumor spheroid

**Figure 8:**
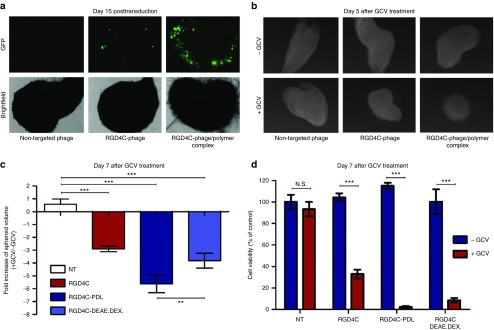
**Efficacy of the hybrid phage/polymer complexes in 9L tumor spheroid models. (a**) GFP expression in the spheroid tumor model. The spheroids were treated with phage/polymer complexes, RGD4C-phage or control non-targeted phage carrying the *GFP* reporter gene. Representative images showing GFP expression in the spheroids were taken at day 15 post-transduction. **(b**) Phase contrast images showing the size of the spheroids following transduction with non-targeted phage, RGD4C-phage or RGD4C-phage/polymer complex carrying the *HSVtk* gene. GCV was added daily and images taken at day 5 following addition of GCV. (**c**) Measurement of volumes of the spheroids in b. (**d**) Evaluation of cell viability in the spheroids at day 7 post-GCV treatment using the CellTiter-Glo cell viability assay.

The *in vitro* tissue spheroids were set up to simulate 3D tumors. 3D spheroids are considered valid models to recapitulate features of tumor microregions, avascular regions, or micrometastases.^25,26^ Most of the tumors are solid and the avascular regions prevent vectors from penetrating into the center of tumors.^27,28^ *In vitro* 3D tumor spheroid model was established to imitate the avascular regions in order to evaluate and confirm the efficacy of gene therapy by the targeted RGD4C-phage/polymer complexes. We first assessed efficacy of gene transfer using phage carrying the *GFP* reporter gene to allow microscopic imaging of GFP expression within the 3D model of 9L tumor spheroids. Phage was added to the spheroids and GFP expression was monitored over a long-time period to allow detectable gene expression by the RGD4C-phage in the spheroids. While the targeted RGD4C-phage showed minimal GFP expression in the spheroids at day 15 post-transduction, treatment with the RGD4C-phage/polymer complexes yielded dramatic increase in GFP expression (**Figure 8a**). These data also show that the 3D spheroid models can overcome the limitation of cell confluence in 2D monolayers and allow monitoring of gene expression over a longer time course after phage vector transduction. Similar results were obtained with the 3D spheroid models of the human LN229 and MCF-7 brain and breast cancers, respectively (**Supplementary Figure S6**).

Next, application of *HSVtk*/GCV suicide gene therapy resulted in pronounced regression of the 9L spheroid volumes by the targeted RGD4C-phage/polymer complexes upon GCV treatment compared with RGD4C-phage alone (**Figure 8b**,**c**). Subsequently, measurement of cell viability in the spheroids showed that the targeted RGD4C-phage/polymer complexes achieved higher tumor cell killing, 98% for RGD4C-PDL and 91% RGD4C-DEAE.DEX, than the targeted RGD4C-phage alone that induced 67% cancer cell killing (**Figure 8d**).

These findings clearly establish that combination of bacteriophage with cationic polymers greatly increase its potential as a gene therapy vector.

## Discussion

Phage nanobiotechnology, initially evolved from phage display technology, has increasingly expanded its applications in diverse disciplines.^29^ Yet, despite some attractive features, bacteriophage viruses are still considered poor vectors for gene transfer, and this limits their application in a broad range of disciplines, such as cancer gene therapy, DNA vaccine delivery, and nanomedicine. However, we have previously reported that phage gene transfer efficacy can be improved by combining bacteriophage with the attributes of animal viruses. This was demonstrated by the incorporation of the genetic *cis*-elements (inverted terminal repeats) from the animal virus AAV2 into the phage genome, resulting in an altered transgene cassette and subsequent enhanced gene delivery efficacy.^16^ Such genetic improvements only become relevant following transfer of the genome to the nucleus. Therefore, the attachment, entry, and intracellular trafficking of the vector remain rate-limiting. We have reported here a highly effective way to improve transduction efficiency of phage vectors. One factor that seems to impede binding is the electrostatic repulsion between the negative charges normally carried by both phage and cell membranes. We have counteracted this by complexing phage with cationic polymers. This novel bacteriophage-derived complex platform is thus based on a combination of genetic and chemical modifications.

The use of polymer coating to modify the properties of nanoparticles is well documented. It has been reported that this increases cell attachment and improves solubility and stability of the particle.^30^ Incorporation of synthetic polymers in eukaryotic viral vectors has also been shown to be beneficial. For instance, the use of polymers to modify the surface of systemically administered adenoviral vectors has been shown to increase anti-tumor activity.^31,32^ Complexation of retroviruses with polymers similarly enhances transduction efficiency.^33,34^ The approach has also been positively applied to AAV2^35^ and baculoviral vectors.^36,37^

We have shown that addition of certain types of cationic polymers to the genetically modified phage at an appropriate ratio leads to the formation of self-assembled complexes that can greatly improve transgene expression both in cell monolayers and in a 3D model that allows cells to grow as spheroids.^38^ The inefficiency of the RGD4C-phage to transduce the tumor spheroids could be associated with limited diffusion of bacteriophage through the extracellular matrix (ECM). Recently, we demonstrated that one limitation of RGD4C-phage transduction efficiency is its weak diffusion and spread though the extracellular matrix (data not shown). The observed surface transduction of the spheroids by the hybrid phage/polymer could also be associated with limited diffusion. The large size of hybrid aggregates could also reduce their diffusion within the spheroids and contribute to surface transduction.

The transduction of mammalian cells by such phage/polymer complexes remains specific and targeted, and occurs through binding of the RGD4C ligand to the α_v_ integrin receptors. Formation of complex prior to its addition to cells seems to be necessary to improve transduction efficiency. Decreased transgene expression is observed at very high polymer concentrations, but this is not related to any detectable cytotoxic effect; it might be due to the formation of larger aggregates that reduce the efficacy of cellular internalization.^39^ It may also be possible that excess of polymers do not integrate into the complex and subsequently affect interaction of the phage with the cells or cause a diminished or competitive polymersome–phage interaction. Nevertheless, the transduction efficiency of the complex, at very high polymer doses, remains more efficient than that of the uncomplexed RGD4C-phage. Finally, it is important to note that the drop in transduction efficiency of the phage/polymer complexes, at high polymer amounts, is associated with a decrease in positive values of zeta potential.

We have observed that accessibility of the uncomplexed phage vector for the cell surface is weak. The negatively charged amino acid residues (Glu2, Asp4, and Asp5) of the major coat protein pVIII are responsible for an overall large negative charge on uncomplexed phage particles.^40^ This could be repelled to some extent by a similar negative charge on the cell membranes of target cells. The addition of the positively charged polymers generated an overall positive charge that greatly improved phage availability on the cell surface.

In addition to the charge, changing and increasing the size of phage/polymer complexes leads to generation of aggregates. These large aggregates might contribute to better phage access and setting on the cell surface,^41^ leading to improved transduction efficiency as long as the polymer dose used does not exceed the optimal polymer dose that results in maximum transduction efficiency. Indeed, confocal imaging revealed that most of the phage localized on the cell surface is in large aggregates. While no polymer effect was observed on cellular internalization of phage, the increased buffering capacity of the phage/polymer complexes and inhibition of their transduction efficiency by bafilomycin A1 give evidence of facilitated escape from the endosomal-lysosomal degradative pathway, thus increasing transduction efficiency. Consistently, we recently reported the endosomal-lysosomal sequestration of RGD4C-phage-derived vectors as a major intracellular limitation to gene delivery.^20^ Previous studies also reported the capacity of cationic polymers to enhance endosomal escape of vectors.^23,24^ Although combination with polymers does not affect phage internalization, it is possible that better cell accessibility of the phage/polymer aggregates increases the availability of phage, overtime, to the cell surface. This characteristic could explain the augmented gene expression achieved by the RGD4C-phage/polymer complexes overtime as compared with RGD4C-phage that reaches saturation of gene expression within a few days.

In brief, our findings suggest that the cationic polymers may bestow an advantage in phage-mediated gene delivery by altering the fate of the bacteriophage capsid through improving the phage cell accessibility and intracellular trafficking. It is noteworthy to mention that although the tumor- targeted adeno-associated virus/phage (RGD4C-AAVP) vector has an altered transgene cassette,^16^ its packaging capsid is similar to that of the conventional RGD4C-phage vector, explaining the comparable effects yielded by the cationic polymers on both RGD4C-phage and RGD4C-AAVP vectors (**Supplementary Figure S7**).

We have also demonstrated the potential of this newly developed phage/polymer vector for clinical applications. It significantly improves the transfer of the suicide *HSVtk* gene, which is used in conjunction with the prodrug GCV for cancer gene therapy. The enzyme thymidine kinase phosphorylates the prodrug GCV to GCV monophosphate, which is then further phosphorylated to the cytotoxic GCV triphosphate, leading to programmed cell death via the inhibition of DNA polymerase. *HSVtk* gene transfer using the hybrid RGD4C-phage/polymer complex followed by the addition of GCV led to complete eradication of cancer cells grown as 2D monolayers or 3D tumor spheroid model. It may be significant that cancer cells have a higher expression of anionic molecules such as phosphatidylserine^42,43^ and *O*-glycosylated mucins^44,45^ than normal cells, resulting in a net negative charge of cancer cell membranes rendering them more attractive to cationic molecules. Our studies also support the proof-of-concept that taking advantage of fundamental differences existing between cell membranes of malignant cells and membranes of normal cells should benefit the design of gene delivery vector systems for improved efficacy of cancer gene therapy.

A number of previous studies have shown that cationic vectors can be combined with receptor-targeting ligands capable of guiding DNA into eukaryotic cells via receptor-mediated mechanisms. Complexes of ligands and cationic vectors are taken up by various cells and exhibit high transfection activity. Both the uptake and transduction efficiency correlate with the expression of receptors.^46^ For instance, conjugates of sugar moieties (*e.g.*, lactose or galactose) with polylysine target polyplexes to asialoglycoprotein receptors of hepatocytes.^47,48^ In addition, transferrin ligand was used to form transferrin–polylysine conjugates that delivered plasmid DNA to various cells.^49^ There have also been efforts to combine antibodies to polylysine polyplexes allowing efficient leukemia-specific cell internalization.^50^ Therefore, while some published papers suggest that a targeting ligand is ineffective with positive charged particles, other studies clearly show that targeting ligands function effectively when combined with positively charged particles. That being said, the targeting ligand efficiency seems to be dependent on the nature of the positively charged material used. Also, while some studies reported inefficiency of cationic polymers *in vivo*, other reports showed that cationic polymers such as polylysine enhanced gene delivery *in vivo*, following intravenous administration, when combined with viral vectors such as AAV2.^35^

In conclusion, we have presented a novel strategy to advance phage-targeted gene transfer by modifying the ζ-potential of bacteriophage to generate positively charged phage surfaces. Phage/polymer complexes were generated by combining M13 filamentous phage with cationic polymers. Our findings show that combination of phage with cationic polymers results in substantial enhancement of gene transfer by bacteriophage in 2D tissue culture and in a 3D multicellular tumor spheroid model. Additionally, the phage/polymer hybrid system is safe and preserves the tumor-targeting property of phage, resulting in cancer cell killing upon treatment with bacteriophage carrying the *HSVtk* cytotoxic gene and GCV. Importantly, we showed that this new complex can target several cancer cell types. This proof-of-concept study successfully shows that the limitations of bacteriophage as vectors for gene transfer into mammalian cells can be overcome. Future preclinical studies to assess efficacy of the phage/polymer complexes *in vivo* in tumor-bearing animals will be necessary to characterize these hybrid complexes, paving the way toward future clinical trials of phage gene therapy in cancer patients.

## Material and methods

*Construction and production of bacteriophage vectors.* To generate bacteriophage-derived vectors for targeted gene delivery, the phage was genetically manipulated to display copies of the RGD4C tumor homing peptide, on the pIII minor coat protein, and to carry a mammalian gene cassette encoding a cytomegalovirus promoter-driven transgene expression. Phage viral particles were amplified, isolated, and purified from the culture supernatant of host bacteria (*Esherichia coli* K91), as we previously reported.^15^ Phage viruses were sterile-filtered through 0.45-µm filters, then titrated using a qNano particle analyzer (IZON Science, Oxford, UK) based on a coulter technique also known as resistive pulse sensing, and expressed as mg/ml.

*Preparation of hybrid bacteriophage/polymer complexes.* Cationic polymers at desired concentrations were added to phage vector preparations, mixed gently, and incubated for 15 minutes at room temperature to form complexes. ζ-potential measurements were conducted using ZetaPALS (Brookhaven Instruments Corporation, NY) based on electrophoresis in 1 mmol/l KCl electrolyte solution. The pH dependency of ζ-potential was measured by changing the pH of the electrolyte solution through the titration of 0.1N HCl or NaOH. Size distribution was also evaluated using a qNano analyzer.

*Cell culture.* HEK293 cell line was purchased from American Type Culture Collection. Human M21 melanoma cells were provided by Dr David Cheresh (University of California, La Jolla). Human LN229, SNB19, and the rat C6 gliomas were provided by Dr Nelofer Syed (Imperial College London, UK). The rat 9L glioblastoma cells were a gift from Dr Hrvoje Miletic (University of Bergen, Norway). The MCF-7 cell line was from the Cancer Research UK. The mouse C_2_C_12_ myoblast cell line was provided by Dr Francesco Muntoni (University College London, UK). All these cell lines were maintained in a humidified atmosphere of 37 °C in a 5% CO_2_ and cultured in Dulbecco's Modified Eagle's Medium (Sigma, Dorset, UK) supplemented with 10% fetal bovine serum (Sigma), penicillin (100 units/ml, Sigma), streptomycin (100 µg/ml, Sigma), and l-glutamine (2 mmol/l, Sigma). The C_2_C_12_ cells were grown in 20% fetal bovine serum.

*Internalization assay.* Cells were treated with vectors for 4 hours at 37 °C, then placed on ice to stop endocytosis and washed three times with phosphate buffered saline (PBS) to remove unbound vectors. Surface bound vectors were removed by trypsinization after which cells were pelleted by centrifugation at 2,000 rpm for 5 minutes and fixed in 4% paraformaldehyde (PFA) for 10 minutes at room temperature. Untreated cells were used as negative controls. To detect internalized phage-derived vectors, cells were blocked with 0.1% saponin in 2% bovine serum albumin in PBS for 30 minutes followed by staining with rabbit anti-fd-phage antibody (diluted 1:1,000) in 0.1% saponin in 1% bovine serum albumin-PBS for 1 hour at room temperature. Cells were pelleted and re-suspended three times in 0.1% saponin in 1% bovine serum albumin-PBS, then incubated with goat anti-rabbit AlexaFluor-647 (diluted 1:500) for 1 hour at room temperature. Finally, cells were washed twice with 0.1% saponin-PBS and re-suspended in PBS before analysis.

Fluorescence-activated cell sorting analysis was carried out using a FACscalibur Flow cytometer (BD Biosciences, Oxford, UK) equipped with an argon-ion laser (488 nm) and red-diode laser (635 nm). The mean fluorescence intensity was measured for at least 10,000 gated cells per triplicate well. Results were analyzed using Flojo (TreeStar) software.

*Endosome buffering capacity measurements.* The acid–base titration method was used to determine the endosome buffering capacities of the phage/polymer complexes prepared to their optimized ratios in sterile water to a total volume of 20 ml and the pH adjusted to 10 by NaOH. Subsequent additions of HCl were used to titrate the solution to pH 3 while changes in pH were recorded using a pH meter. Titrations of NaCl solution, polymer solution, and phage were used as controls. The natural endosome of pH r 7.0–4.0 was used to calculate the endosome buffering capacity of the phage/polymer complexes.

*In vitro cell transduction by phage-derived vectors.* Cells were seeded into 48-well plates and grown for 48 hours to reach 60–80% confluence. The hybrid phage/polymer complexes, prepared at optimal ratios, or phage vector alone were applied to cells in serum-free media, followed by 4-hour incubation at 37 °C. The medium was then replaced with fresh serum-containing medium and cells incubated at 37 °C to allow transgene expression. Determination of cell transduction efficacy by the phage vectors was performed using phage carrying the *Luc* and *GFP* reporter transgenes. The *Luc* reporter gene expression in transduced cells was determined with The Promega Steady-glo luciferase assay kit following the manufacturer's protocol and quantified using a Promega plate reader, then normalized to 100 µg protein levels as determined by the Bradford assay and data presented as relative luminescence units (RLU) per 100 µg of protein. For competition experiments, cells were first incubated with the RGD4C-expressing phage for 1 hour in serum-free medium and then transduced with the targeted RGD4C-PDL or RGD4C-DEAE.DEX complexes. Cell viability was determined by CellTiter-Glo cell viability assay kit following the manufacturer's protocol and quantified using a Promega plate reader.

*Determination of tumor cell killing in vitro.* 9L cells were seeded in 48-well plates and incubated for 48 hours to reach 60–80% confluence. Next, cells were transduced with the hybrid complex or phage vector alone carrying the *HSVtk* gene. GCV was added to cells (10 µmol/l) at day 3 post-transduction and renewed daily. Viable cells were monitored under microscope and cell viability was measured at days 1, 3, and 5 post-GCV treatment using the CellTiter-Glo cell viability assay kit.

*Model of the 3D multicellular tumor spheroid culture and treatment.* To prepare multicellular tumor spheroid, 200 μl of cell suspension (0.5 × 10^4^ cell/ml) was seeded into a 96-well ultra-low attachment surface plates (Corning, Nottingham, UK). After 48 hours of incubation, a multicellular spheroid was spontaneously formed in each well. Then, after removing 100 μl of media, the hybrid phage/polymer complexes, prepared at optimal ratios, or phage vector alone in 100 μl were applied to each well for transduction. After 24 hours, the medium was replaced with 200 μl of medium containing 10% serum. The medium was replaced, every 3 days, by fresh medium containing 10% serum. *GFP* gene expression was then evaluated using a fluorescent microscope.

*Confocal microscopy.* Cells were seeded on 18 mm^2^ coverslips in 12-well plates. To stain for α_v_ integrins, cells at ~50–60% confluence were washed with PBS and fixed in 4% paraformaldehyde for 15 minutes at room temperature. For phage staining, cells were incubated with phage vectors for 4 hours at 37 °C, followed by washing with PBS and fixation with 4% paraformaldehyde. Following fixation, cells were treated for 5 minutes with 50 mmol/l ammonium chloride to quench free aldehyde groups from fixation, permeabilized with 0.2% Triton X-100, washed, and blocked with PBS containing 2% bovine serum albumin. Next, cells were incubated with primary antibodies: rabbit anti-α_v_ integrin (diluted 1:50), rabbit anti-phage (1:1,000) for 1 hour, then with secondary AlexaFluor-conjugated antibodies (diluted 1:750) with/without DAPI (diluted 1:2,000) for 1 hour at room temperature. Finally, cells were mounted in Mowiol mounting medium (prepared in-house). Images were acquired with a Leica SP5 confocal microscope.

*Statistical analysis.* Statistical analyses were performed using GraphPad Prism software (version 5.0). Error bars represent standard error of the mean. *P* values were generated by analysis of variance and denoted as follows: *P < 0.05, **P < 0.01, and ***P < 0.001.

**SUPPLEMENTARY MATERIAL** **Figure S1**. Targeted RGD4C-phage/polymer complexes enhance phage-mediated gene delivery in various cancer cell lines. **Figure S2**. Hybrid phage/polymer complexes boost gene transfer efficiency of phage in the human non-tumorigenic HEK23 cells. **Figure S3**. Expression of GFP in HEK293 cells by the phage/polymer complexes. **Figure S4**. Separate applications of polymer and phage on target cells have no effect on gene transfer. **Figure S5**. Effect of cationic polymers on phage internalization. **Figure S6**. Efficacy of the hybrid phage/polymer complexes in LN229 and MCF-7 tumor spheroids. **Figure S7**. Cationic polymers produce similar fold increase of gene delivery by targeted RGD4C-AAVP vector.

## Supplementary Material

Supplementary Figure S1 Targeted RGD4C-phage/polymer complexes enhance phage-mediated gene delivery in various cancer cell lines.

Supplementary Figure S2 Hybrid phage/polymer complexes boost gene transfer efficiency of phage in the human non-tumorigenic HEK293 cells.

Supplementary Figure S3 Expression of GFP in HEK293 cells by the phage/polymer complexes.

Supplementary Figure S4 Separate applications of polymer and phage on target cells have no effect on gene transfer.

Supplementary Figure S5 Effect of cationic polymers on phage internalization.

Supplementary Figure S6 Efficacy of the hybrid phage/polymer complexes in LN229 and MCF-7 tumor spheroids.

Supplementary Figure S7 Cationic polymers produce similar fold increase of gene delivery by targeted RGD4C-AAVP vector.
