# Supplementary figures and images for: Hybrid Nanomaterial Complexes for Advanced Phage-guided Gene Delivery
Source: Mol Ther Nucleic Acids. 2014 Aug 12;3(8):e185–. doi: 10.1038/mtna.2014.37 (PMC4221597; doi:10.1038/mtna.2014.37)

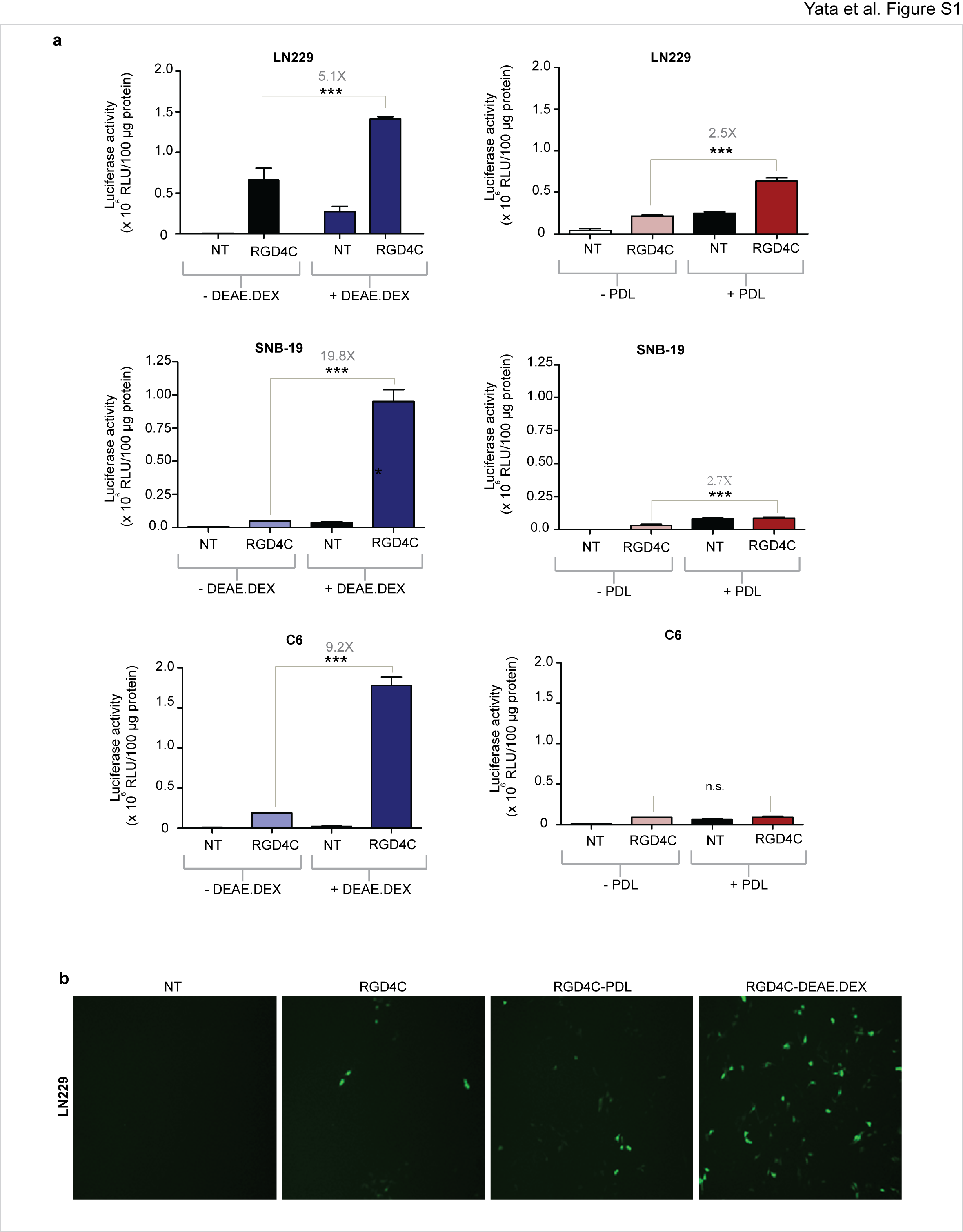

Supplement: Supplementary Figure S1 — Targeted RGD4C-phage/polymer complexes enhance phage-mediated gene delivery in various cancer cell lines. [file mtna201437x1.tiff]

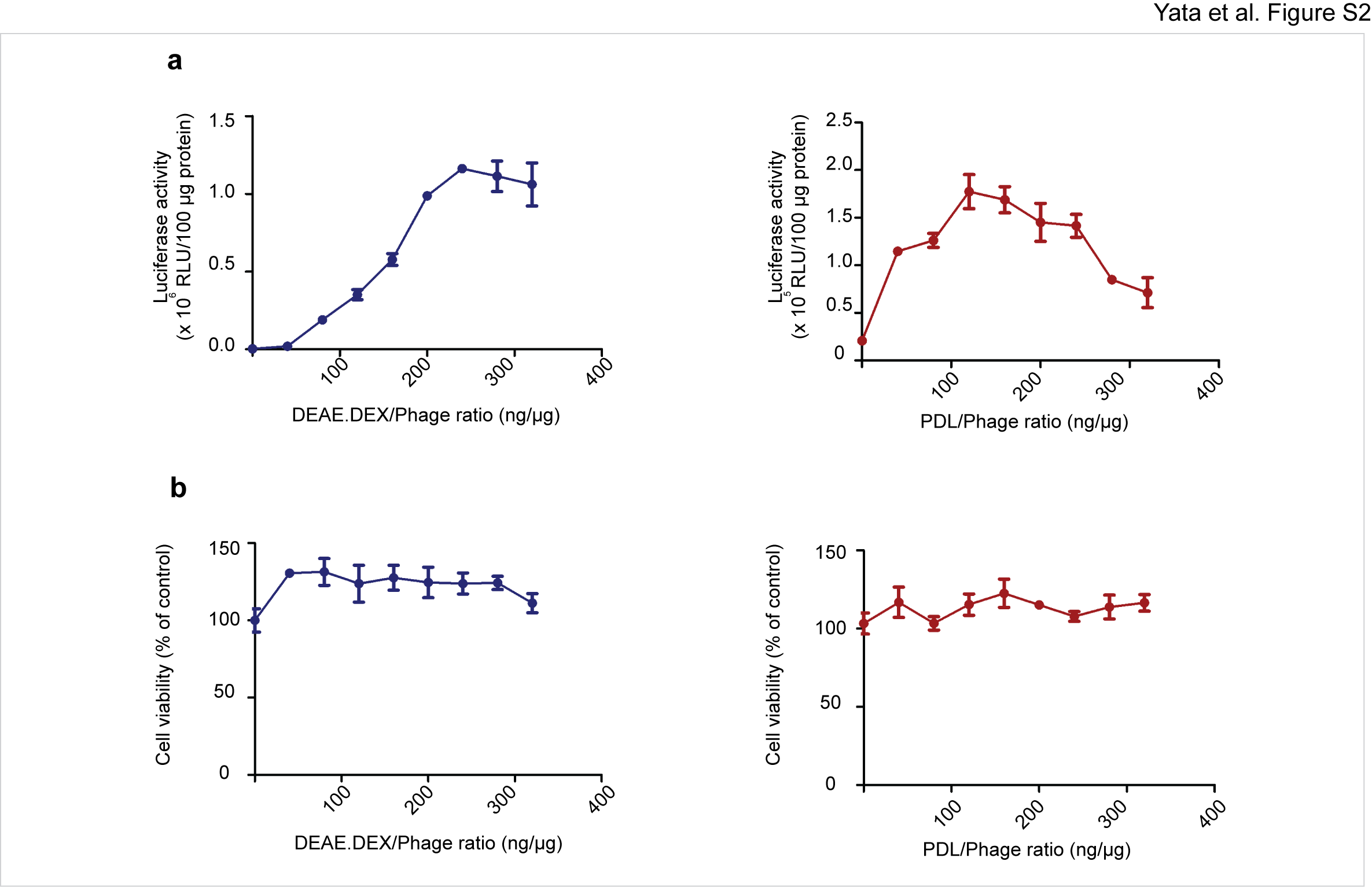

Supplement: Supplementary Figure S2 — Hybrid phage/polymer complexes boost gene transfer efficiency of phage in the human non-tumorigenic HEK293 cells. [file mtna201437x2.tiff]

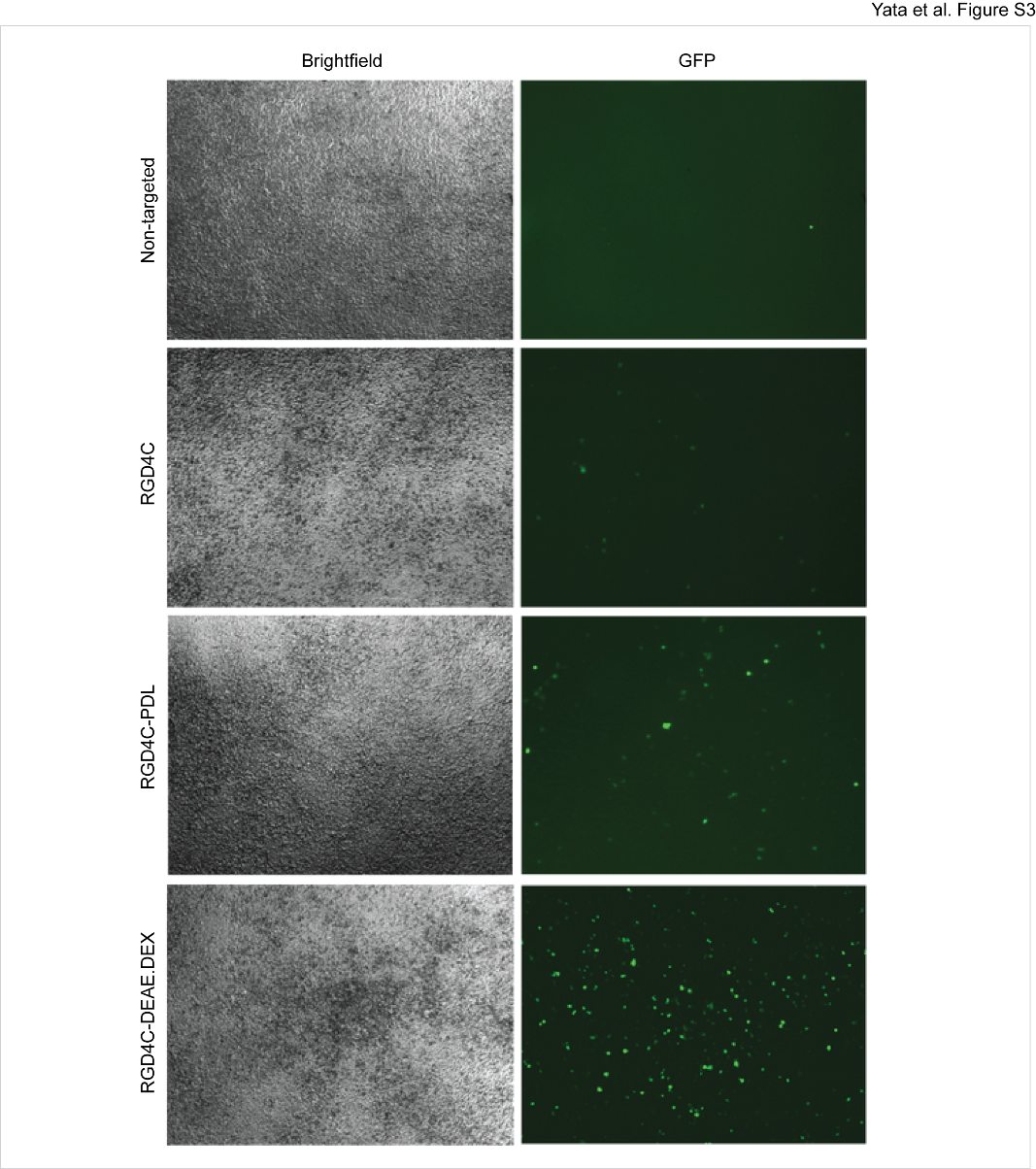

Supplement: Supplementary Figure S3 — Expression of GFP in HEK293 cells by the phage/polymer complexes. [file mtna201437x3.tiff]

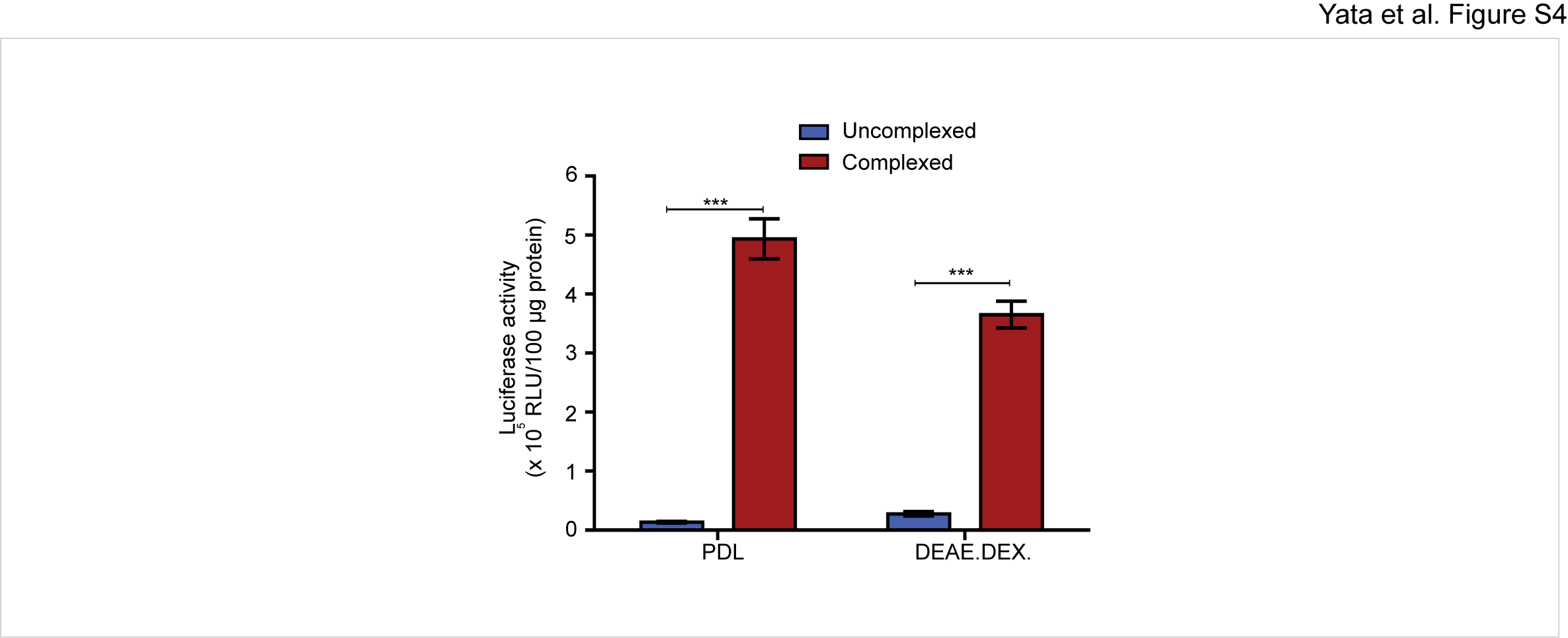

Supplement: Supplementary Figure S4 — Separate applications of polymer and phage on target cells have no effect on gene transfer. [file mtna201437x4.tiff]

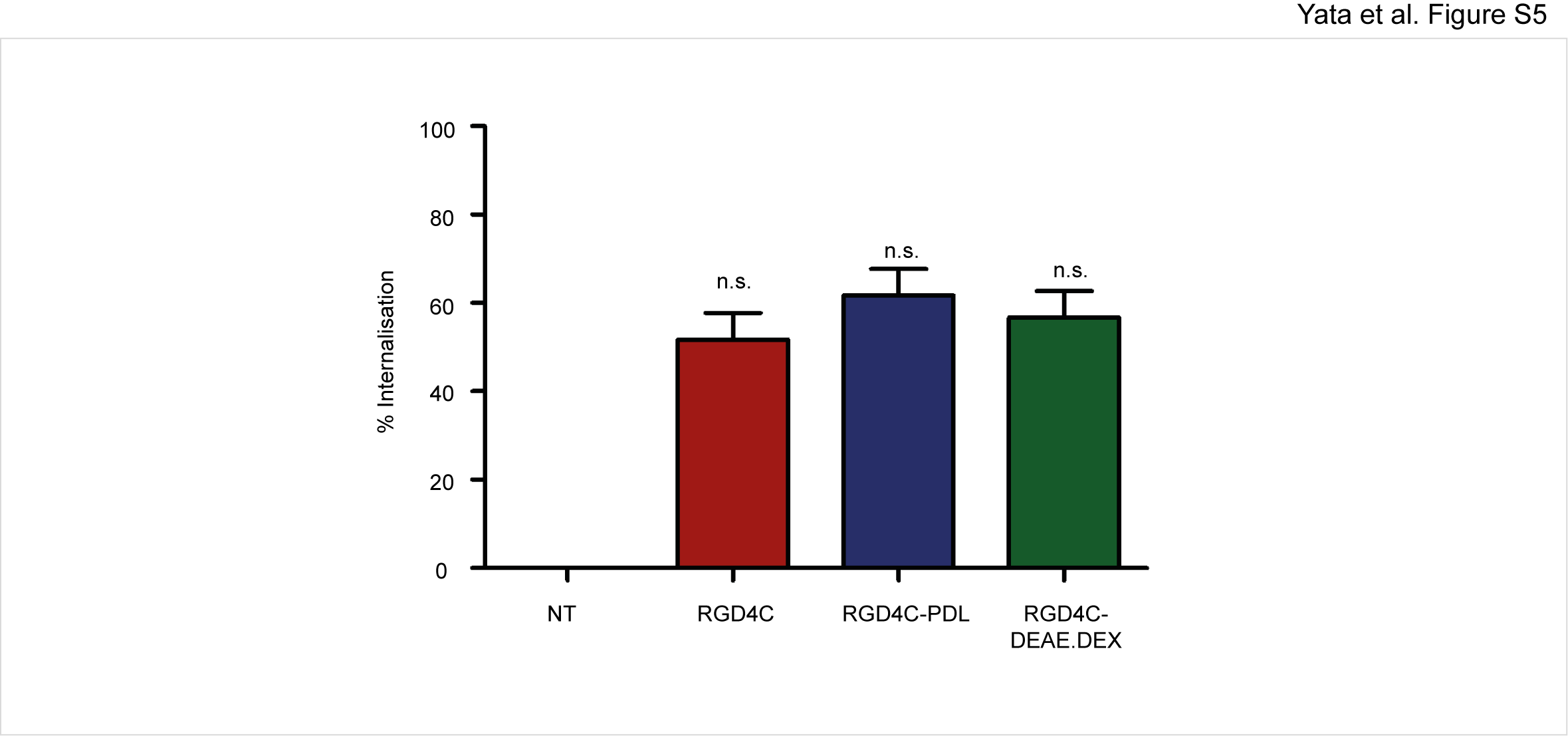

Supplement: Supplementary Figure S5 — Effect of cationic polymers on phage internalization. [file mtna201437x5.tiff]

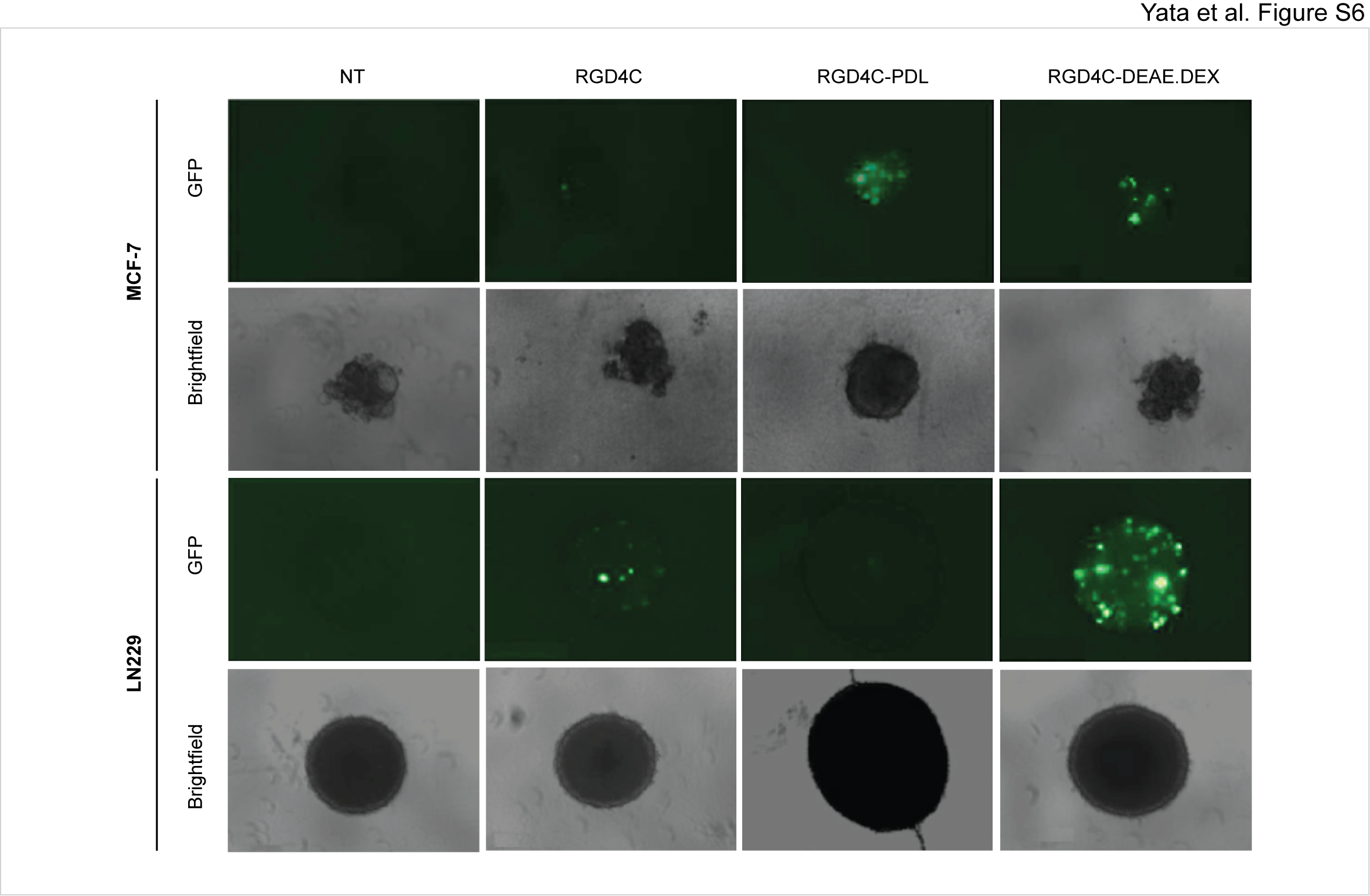

Supplement: Supplementary Figure S6 — Efficacy of the hybrid phage/polymer complexes in LN229 and MCF-7 tumor spheroids. [file mtna201437x6.tiff]

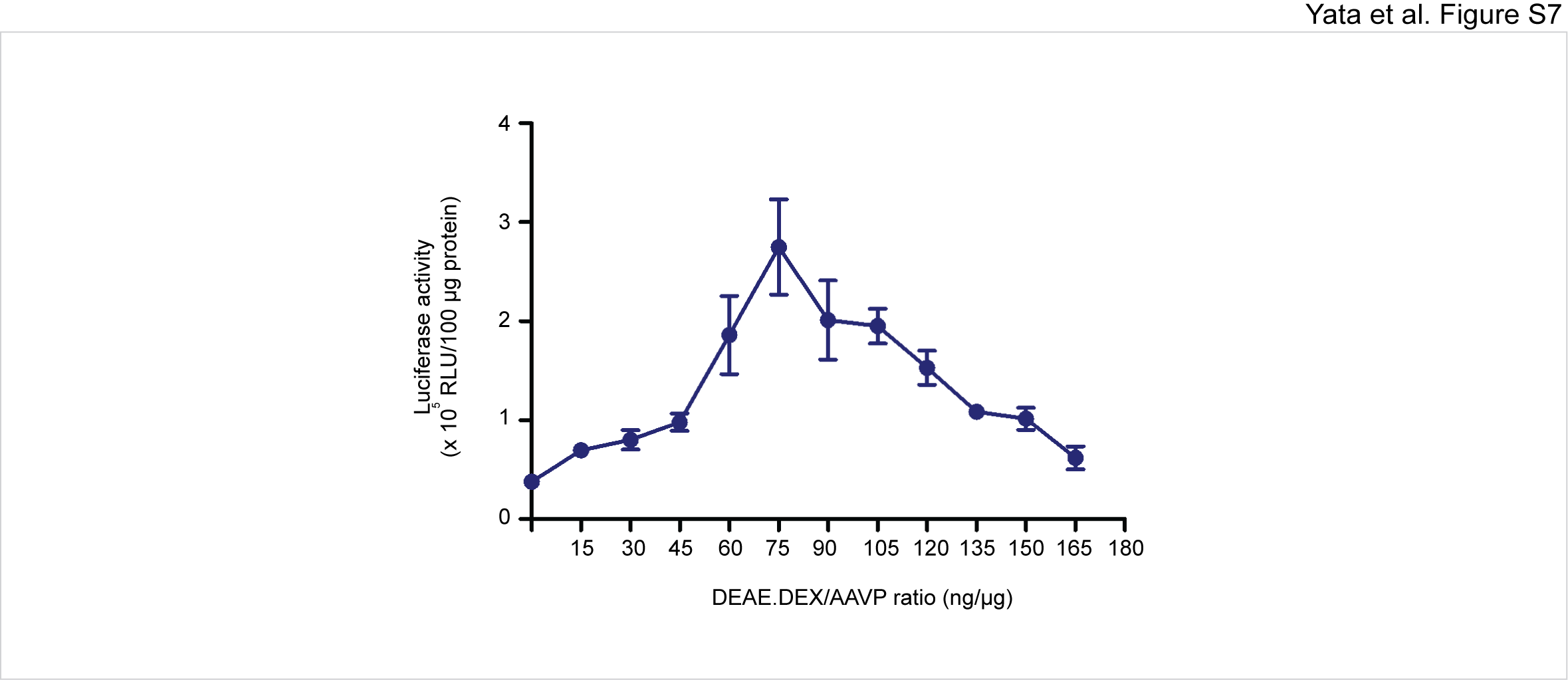

Supplement: Supplementary Figure S7 — Cationic polymers produce similar fold increase of gene delivery by targeted RGD4C-AAVP vector. [file mtna201437x7.tiff]
